# Molecular Mechanisms Modulating the Phenotype of Macrophages and Microglia

**DOI:** 10.3389/fimmu.2017.01520

**Published:** 2017-11-10

**Authors:** Stephanie A. Amici, Joycelyn Dong, Mireia Guerau-de-Arellano

**Affiliations:** ^1^School of Health and Rehabilitation Sciences, Division of Medical Laboratory Science, College of Medicine, Wexner Medical Center, The Ohio State University, Columbus, OH, United States; ^2^McCormick School of Engineering, Division of Biomedical Engineering, Northwestern University, Evanston, IL, United States; ^3^Institute for Behavioral Medicine Research, The Ohio State University, Columbus, OH, United States; ^4^Department of Microbial Infection and Immunity, The Ohio State University, Columbus, OH, United States; ^5^Department of Neuroscience, The Ohio State University, Columbus, OH, United States

**Keywords:** macrophages, microglia, central nervous system, inflammation, molecular, microRNA, metabolism

## Abstract

Macrophages and microglia play crucial roles during central nervous system development, homeostasis and acute events such as infection or injury. The diverse functions of tissue macrophages and microglia are mirrored by equally diverse phenotypes. A model of inflammatory/M1 versus a resolution phase/M2 macrophages has been widely used. However, the complexity of macrophage function can only be achieved by the existence of varied, plastic and tridimensional macrophage phenotypes. Understanding how tissue macrophages integrate environmental signals *via* molecular programs to define pathogen/injury inflammatory responses provides an opportunity to better understand the multilayered nature of macrophages, as well as target and modulate cellular programs to control excessive inflammation. This is particularly important in MS and other neuroinflammatory diseases, where chronic inflammatory macrophage and microglial responses may contribute to pathology. Here, we perform a comprehensive review of our current understanding of how molecular pathways modulate tissue macrophage phenotype, covering both classic pathways and the emerging role of microRNAs, receptor-tyrosine kinases and metabolism in macrophage phenotype. In addition, we discuss pathway parallels in microglia, novel markers helpful in the identification of peripheral macrophages versus microglia and markers linked to their phenotype.

## Introduction

Macrophages in the central nervous system (CNS) play important homeostatic and immune defense roles (1). While microglia originate from early yolk sac myeloid progenitors and become self-regenerating CNS-resident cells (2–4), macrophages originate from peripheral blood monocytes. Microglia are essential for appropriate synaptic pruning during development (1). During steady state condition, microglia also facilitate learning and memory and remove cellular or other debris. Upon CNS infections and injury, microglial activation and peripheral macrophage recruitment and activation occur. Both macrophages and microglia have the capacity to recognize pathogens or injured cells, activating phagocytic, antigen-presenting and cytokine/chemokine secretion functions that modulate immunity and mediate pathogen or cellular debris elimination (1). Macrophages and microglia also contribute to resolution stages of inflammation and tissue regeneration *via* switching to anti-inflammatory cytokine patterns, promoting intercellular matrix synthesis and angiogenesis. The complexity of macrophage function is mirrored by the existence of varied, plastic and multilayered macrophage phenotypes *in vivo* (5). However, for simplicity, a model of inflammatory/classical M1 and resolution/alternatively activated M2 macrophages has been widely used.

Understanding the molecular programs that define inflammatory versus resolution phenotypes provides the opportunity to target and modulate these cellular programs to control the excessive inflammation typical of chronic inflammatory CNS conditions such as Multiple Sclerosis (MS) and CNS injury. In recent years, our understanding of how environmental signals are integrated into macrophage phenotype has greatly advanced. The classic roles of NOTCH, PI3K/AKT, MYC, PPAR, and interferon regulatory factors (IRFs) in macrophage polarization have been further established while prominent roles for metabolism, microRNAs (miRNAs) and receptor-tyrosine kinases (RTKs) are now clear. The ability to distinguish microglia from CNS macrophages and inflammatory vs. resolution macrophages has greatly advanced with the discovery of new markers. In this review, we discuss these findings and present the current understanding in the field of molecular mechanisms and markers of inflammatory versus resolution macrophages, as well as therapeutic implications of macrophage modulation for the CNS autoimmune disease MS.

## Macrophages and Microglia: Similar but Not the Same (Developmental Origin, Functions, and Markers)

Microglia and macrophages have many functions in common. They both help to maintain homeostasis during embryogenesis and into adulthood (6–8). Additionally, both cells are sentinels in their respective environments, scanning for foreign invaders and pathogens (9–12). Both cell types also differentiate into a spectrum of proinflammatory to proregenerative subsets in response to injury or insult (13, 14). In addition to the roles microglia play in fighting infection and clearing debris *via* phagocytosis, microglia are also important in neuronal proliferation and differentiation and the formation and pruning of synaptic connections in neuronal networks (15, 16). Based on the specific genes expressed in microglia and the subset of functions unique to microglia, one can postulate that other tissue-specific macrophages have roles exclusive to their tissue that monocyte-derived macrophages cannot replace.

Monocyte-derived CNS macrophages and microglia have similar morphologies and phagocytic functions but their origins are distinct. Until a short time ago it was believed that solely circulating monocytes replenish tissue macrophage populations, including those in the CNS, but this view is now rejected based on new reports in the literature (17, 18). Although bone marrow derived monocytes can enter tissues such as the CNS and differentiate into macrophages, microglia and other tissue macrophages are now thought to originate most exclusively from earlier embryonic progenitors (19). Embryonic hematopoiesis consists of three main waves, namely primitive, transient definitive and definitive hematopoiesis. Primitive hematopoiesis originates from yolk sac blood islands around embryonic day (E)7, yielding progenitors as early as E7.5 (19). The transient definitive hematopoiesis wave starts around E8 when hemogenic endothelium develops, producing erythromyeloid precursors (EMPs) (19). Upon establishment of circulation starting at E8.5, EMPs migrate to the fetal liver where they support definitive hematopoiesis (19). EMPs will also eventually migrate and support bone marrow hematopoiesis in the adult.

Three models of fetal microglia and tissue macrophage ontogeny have been proposed (19). Two models favor the view that most microglia but few of other tissue macrophages derive from the early wave of primitive hematopoiesis in the yolk sac (20, 21). The remaining model instead supports the view that EMPs from transient definitive hematopoiesis give rise to most microglia and other tissue macrophages (22). All these models are all in agreement on the embryonic origin of microglia, with little or no contribution from monocytes. Sublethally irradiated C56BL/6 CD45.2^+^ newborn mice reconstituted with hematopoietic cells isolated from CD45.1^+^ congenic mice had 95% microglia were of host origin (CD45.2^+^ cells gated on CD11b^+^CD45^int^, then Ly6G^-^F4/80^+^) 3 months after transplant, while over 30% of circulating leukocytes were of donor origin (2). These data support the idea that microglia are a distinct population not populated/replenished by circulating monocytes. Kierdorf and colleagues added to our knowledge by identifying the earliest yolk sac progenitors with the potential to become microglia to be CD45^−^ c-Kit^+^ erythromyeloid precursors (EMPs), and these differentiated into Iba-1^+^ Cx3cr1^−^ cells with microglial-like morphology (3). Two transcription factors important in driving EMPs to differentiate into microglia and CNS macrophages are PU.1 and IRF-8. *Pu.1* gene deficient animals lacked microglia completely, while mice-lacking the *Irf8* gene had significantly reduced numbers of microglia (3). Upon analysis of the yolk sac progenitors, they found that PU.1 is necessary for the initial transition from EMPs (c-KIT^+^) to early microglial precursors (CD45^+^ c-KIT^lo^ CX3CR1^−^); whereas IRF-8 acts downstream of PU.1 and plays a role in the transition from early to mid-stage microgliogenesis (CD45^+^ c-KIT^−^ CX3CR1^+^) (3). Another molecule important in shaping microglial development is negative regulator of reactive oxygen species (NRROS, aka LRRC33). *Nrros* gene deficient mice lack normal CD11b^hi^CD45^lo^ microglia, and CX3CR1-driven deletion of *Nrros* leads to impaired expression of *Sall1* (lineage-specific transcription factor important for maintenance in adult microglia) and other microglial genes needed for microglial development and function (23). Interestingly, *Nrros*^−/−^ mice have normal numbers of myeloid progenitor cells in the CNS at E10.5, but the CD11b^hi^CD45^lo^ microglial population was largely absent by E14.5, suggesting NRROS is important in early microglial development.

### Can Microglia Be Differentiated from Peripheral Origin Macrophages?

Discriminating between peripheral macrophages and microglia has been a difficult technical issue. Microglia and macrophages share many markers such as CD11b, F4/80, CX3CR1 and IBA1 (13). High levels of CD45 expression (CD45^hi^) have long been used to discern peripheral macrophages from microglia, which express lower levels of CD45 (24). However, peripheral macrophages may downregulate CD45 once in the CNS or in response to injury (25). CX3CR1 (aka fractalkine receptor) is expressed by microglia throughout development and into adulthood (26). Since it is not expressed by other CNS-origin cells (27), CX3CR1 can be used to detect microglia in naive tissues. During inflammation, however, peripheral macrophages, monocytes and T cells also express CX3CR1 and infiltrate the CNS (28). The use of irradiated *Cx3cr1*–green fluorescent protein (GFP) knock-in mice (27, 28) as recipients of WT bone marrow yields a model in which only microglia express GFP and peripheral macrophages can be detected by use of donor markers. Another model, a tamoxifen-inducible Cre mouse line crossed with a red fluorescent protein (RFP) Cre reporter mouse line (*Cx3cr1*^YFP-CreER/wt^:R26^RFP^), can differentially label microglia and recruited macrophages by pulsing mice with tamoxifen and then following the YFP^+^RFP^+^ labeled cells (29). Macrophages will turnover quickly and lose RFP expression, while microglia will retain RFP expression because they are long-lived. Besides a marker and chemoattraction role, CX3CR1 has an essential role in promoting a resting microglial phenotype and neuroprotection (30). *Cx3cr1*^−/−^ mice had worsened neurologic dysfunction in the EAE model (31). In contrast, post-spinal cord injury (SCI) recovery was enhanced in *Cx3cr1*^−^*^/^*^−^ mice (32), suggesting context and CX3CR1 expression on cells beyond microglia and macrophages influence disease outcomes. Soluble CD163, which is cleaved from CD163 on macrophage/microglia membranes, may also be a marker for MS or for inflammation in general (33–35). In addition, many markers co-expressed by peripheral macrophages and monocytes are present on activated microglia as well. For example, CD169 is a marker for macrophages (13) that was recently identified on early activated microglia in MS and EAE lesions (36). MERTK is another common marker for many tissue specific macrophages including microglia (13).

The most prominent difference between microglia and macrophages appears to be their developmental origin. One marker specific to microglia that does not stain infiltrating peripheral immune cells is TMEM119 (25, 37). TMEM119 protein is expressed on all microglia by postnatal day 14 (P14) and remains expressed in post-sciatic nerve crush injury, LPS injection and optic nerve crush injury (25). Specifically, *Ccr2*^RFP/+^ mice (in which RFP is only expressed in infiltrating monocytes) showed IBA1^+^TMEM119^−^ cells were mostly RFP^+^ and RFP^+^ cells were never TMEM119^+^, suggesting TMEM119 is a stable resident microglia marker that does not recognize infiltrating macrophages. Importantly, TMEM119 is a marker for both mouse and human microglia (25) and is maintained in MS lesional tissue (38). The availability of this marker has revealed that many microglial markers are induced, while macrophage markers are suppressed, in peripheral macrophages that infiltrate the CNS (38). FCRLS is another highly expressed gene specific to murine microglia, but there is no ortholog in humans (39). Another microglial-specific marker P2RY12, a purinergic receptor associated with homeostatic microglia not detected on lymphatic tissue (38), is highly expressed in normal white matter of MS patients. However, as microglia become stimulated in active MS lesions, P2RY12 is sometimes downregulated, while proinflammatory genes such as iNOS and CD86 are upregulated (38), confounding the distinction of microglia from peripheral cells during inflammation. Since another report found that P2RY12 remains elevated after EAE induction (39), this may be a human/mouse model difference. TMEM119 seems to be the most discriminatory of the new markers and has allowed to sort microglia based on their developmental origin. These studies have revealed other genes specific of this population that may lead to additional methods for microglial detection (25).

## Macrophage/Microglia Phenotypes, Function, and Nomenclature

The dual role of macrophages and microglia in promoting inflammation vs. resolution is mediated by distinct gene expression programs and macrophage phenotypes. This inflammatory phenotype is induced by ligation of pathogen recognition receptors (PRRs), such as Toll-like receptors (TLRs), on macrophages to pathogen- or danger-associated molecular patterns (PAMPs or DAMPs) from microbes or damaged/dying cells (40). These signals may be combined with inflammatory cytokines produced by Th1 cells, such as IFN-γ. *In vitro*, this phenotype has been modeled by stimulation of bone marrow-derived macrophages or microglia with LPS (+IFN-γ). Macrophages activated in this manner have long been known as classically activated or M1 macrophages (41). In 2014, in an effort to reach consistency and clarity in the field, novel nomenclature that follows the letter M by a parenthesis enclosing the stimuli used for activation was proposed (42). For example, M1 macrophages stimulated with LPS and IFN-γ are indicated as M(LPS + IFN-γ) while macrophages stimulated with LPS alone would be labeled M(LPS). Macrophages differentiated with GM-CSF, or M(GM-CSF) macrophages, have also been described to have a proinflammatory phenotype (43). This nomenclature is providing an extremely useful standardized tool to communicate macrophage experimental data. In this review, we use this nomenclature when the specific stimulation is known, while the simple M1 vs. M2 notation is used when referring to a general inflammatory vs. resolution/alternatively activated phenotype of macrophages.

Functionally, M1 macrophages are responsible for fighting bacterial infections and adopt a phenotype characterized by microbicidal, antigen-presenting and immune potentiating abilities. This is accomplished by induction of inducible nitric oxide synthase (iNOS, encoded by the *Nos2* gene), which synthesizes microbicidal nitric oxide (NO) in most rodent models (44, 45). It is important to note, however, that iNOS induction does not occur in human macrophages. In addition, M1 macrophages recruit additional cells to the site of infection and bridge innate and adaptive immunity. This is accomplished by induction of chemokines and inflammatory cytokines interleukin (IL)-6, IL-12, IL-1β, IL-23, and TNF-α that recruit immune cells to sites of infection and polarize them to type I responses and by CD80 and CD86 costimulatory molecule expression to prime T cells (42, 46). Our lab has recently characterized CD38 as a marker that is increased in inflammatory murine bone marrow-derived M(LPS + IFN-γ) macrophages and decreased in M2 macrophages compared to untreated M0 macrophages (47). CD38 upregulation is also observed in a sepsis model (47) as well as Experimental Autoimmune Encephalomyelitis (EAE), the mouse model of MS (48). Given that CD38 is a surface marker that allows live cell sorting for downstream applications, it provides an advantage over intracellular markers such as iNOS. Although CD38 is known to be an ectoenzyme that catalyzes conversion of NAD to ADP-ribose and induces calcium signaling inside the cell (49), its exact role in inflammatory phenotype is unknown. However, it appears to play an important role, as CD38 induction by LXR and NAD depletion is necessary to limit bacterial uptake and inflammatory cytokine production (50). Future studies will be necessary to determine whether CD38 plays a similar role in human macrophages. For a listing of current inflammatory phenotype markers in macrophage/microglia, see Table 1. Like macrophages, microglia secrete inflammatory cytokines IL-1β, IL-6, IL-12, and TNF-α when exposed to LPS (+IFN-γ). Similarly, they upregulate iNOS and CD38 (51–56). Although most studies have been done in murine microglia, LPS + IFN-γ also induces M1 phenotype in primary human microglia (57).

**Table 1:** Inflammatory phenotype macrophage/microglia markers.

		M(LPS + IFNγ)	M(IL-4)
Transcription factors	Mouse	pSTAT1	pSTAT6, Irf4

	Human	pSTAT1, IRF1, IRF5	IRF4

Amino acid metabolism	Mouse	iNOS	Arginase-1

	Human	IDO1	

Scavenger receptors	Mouse		Mrc1 (CD206), CD163

	Human		MRC1 (CD206), CD163

Cytokines	Mouse	TNFα, IL6, IL12A, IL23A, IL27	

	Human	TNF, IL1B, IL6, IL12A, IL12B, IL23A	

Others	Mouse	CD38, CD80, CD86, FPR2	RELMα (FIZZ1), CHI3L3 (YM-1), ALOX15, EGR2, c-MYC

	Human	CD40	ALOX15

The evolution from acute inflammation to a resolution phase occurs as initial neutrophils undergo apoptosis and monocytes, which will switch to a resolution/M2 phenotype, predominate in the tissue (58). Key lipid mediators in promoting the resolution phase include classical eicosanoids, phospholipids and sphingolipids, endocannabinoids (eCBs) and specialized proresolving mediators (SPMs) (59). The classical eicosanoids thromboxanes (TX) and prostacyclins antagonize inflammation while phospholipids and sphingolipids such as phosphatidylserine (PtdlSer), when recognized by macrophages, promote M2 switch (60). eCBs such as N-arachidonoylethanolamine (AEA) and N-palmitoylethanolamine (PEA) have immunomodulatory roles, particularly in neuroinflammation (61). Last, but not least, SPMs have major proresolution activities. Main SPMs include lipoxins (LX)A4 and LXB4, resolvins (Rv) RvD1-6 derived from docosahexanoic acid (DHA), RvE1-3 derived from eicosapentanoic acid (EPA), protectin D1 and maresins. In particular, maresins have been shown to shift to a resolving macrophage phenotype (62), which can also be induced by exposure to Th2 cytokines like IL-4 and IL-13, parasites, fungal cells, apoptotic cells, immune complexes, adenosine, or transforming growth factor (TGF)-β (63). *In vitro*, four M2 type macrophages were initially described, corresponding to macrophages stimulated with IL-4 or IL-13 (M2a), IL-1R ligands or immune complexes (M2b), IL-10, TGF-β or glucocorticoid (M2c) and IL-6 and adenosine (M2d) (64). This notation has now been replaced with the M(stimulus) nomenclature that clearly defines the inducing stimulus (42). For, example, macrophages stimulated with IL-4 are indicated as M(IL-4) and pro-M2 macrophages differentiated with M-CSF are called M(M-CSF).

It is currently being debated whether resolution/wound healing macrophages are an evolution of initial inflammatory macrophages under the changing local environment or, rather, they originate from newly recruited peripheral monocytes. Similarly, resolution spectrum macrophages may revert to an inflammatory phenotype if new inflammatory stimuli are encountered (65). Resolution macrophages suppress IL-12 secretion and may secrete anti-inflammatory mediators IL-10, TGF-β, IL-1R antagonist (IL-1RA), and decoy IL-1R II (66). In addition, these macrophages express arginase-1 instead of iNOS, switching arginine metabolism from production of NO to ornithine and polyamines for collagen and extracellular matrix synthesis (67). M2 markers arginase-1, resistin-like alpha (RELMα/FIZZ1), and chitinase 3-like protein 3 (CHI3L3/YM1) are detected in murine but not human macrophages (68–70), although it should be noted that a portion of murine M1 stimulated cells also upregulates arginase-1 (42, 47). Murine M(IL-4), but not M(LPS + IFN-γ), bone marrow derived macrophages (BMDMs) can be identified by flow cytometry detection of the intracellular transcription factor EGR2 (47), which is related to the M2 transcription factor C-MYC. C-MYC (47, 71) and CD206 (41) are M2 markers common to murine and human macrophages. CD169 [aka sialic acid binding Ig-like lectin-1 (SIGLEC-1)] in certain *in vivo* macrophage populations (72) and tyrosine-protein kinase Mer (MERTK) in M2c macrophages (73) have also been identified as markers useful in flow cytometry (13, 74). CD169 and MERTK are also expressed in microglia.

Does macrophage and microglial phenotype modulate neuroinflammation and MS? The hypothesis that an inflammatory phenotype in macrophages or microglia is damaging to the CNS while a resolving phenotype contributes to neuroregeneration was introduced by the Popovich group in the late 2000s (75). They found that while inflammatory macrophage responses cause neurotoxicity, resolving macrophage responses instead promote neuron viability and regenerative growth toward repair (75). Evidence supporting this hypothesis, which has important therapeutic implications, has since accumulated in multiple neuroinflammatory paradigms. We will discuss below the latest evidence for a role of macrophage and microglial phenotype on modulation of CNS neuroinflammation and remyelination in multiple sclerosis and its animal model EAE.

Activated microglia are one of the first observations very early in MS while both monocyte-derived macrophages and activated P2RY12^−/lo^ microglia are found later in active lesions (38). Large numbers of macrophages and microglia, coinciding with myelin breakdown, are a hallmark of the acute MS lesion (76). High oxidative activity and expression of IL-1β and IL-23p19, all characteristic of inflammatory macrophages and microglia, are observed in these lesions. Exacerbated and fast-progressing EAE occurs in mice with a microglial-specific Nr4a1 deficiency which results in increased microglial activation and NO production, consistent with the damaging M1 responses. Neurodegenerative microglia characterized by a TREM2-APOE pathway signature, are generated after neuron phagocytosis, further establishing a neurodegenerative cycle (77). Inflammatory microglia do in turn induce a subtype of inflammatory astrocytes, termed A1, which can no longer sustain neurons and induce neuron and oligodendrocyte cell death. A1 astrocytes are abundant in the CNS of various neurodegenerative diseases, including MS lesions (78).

So, what about resolution? It is unclear what exactly makes an active MS lesion evolve toward resolution. PtdlSer, a phospholipid present in myelin, may play a role. PtdlSer liposomes suppress NO and inflammatory cytokine production in macrophages and *in vivo* treatment ameliorates EAE (79). The early resolving lesion contains instead lipid-laden, aka foamy, macrophages or microglia spread throughout the lesion and forming a layer at the lesion edge. There is no evidence of remyelination in early resolving lesions but examples of remyelination in late resolving lesions are fairly common (80). Interestingly, foamy macrophages or microglia abound within these remyelinated areas (80, 81), as if consistent with a reparative role. Consistent with a beneficial role of resolution macrophages, mice deficient in the M2-promoting factor SOCS3 suffer from chronic and more severe EAE while adoptive transfer or promotion of resolution spectrum macrophages suppresses EAE disease (82–84). Besides being less neurotoxic and chemotactic, M2 macrophages may play an active proregeneration role. Consistent with the latter, a shift from inflammatory to resolving phenotype in microglia and infiltrating macrophages is observed during remyelination (81). The shift to M2 phenotype drives oligodendrocyte differentiation in an activin A-dependent manner (81). Taken together, these findings provide a framework for the importance of inflammatory macrophage and microglial phenotype in driving MS neuroinflammation and the therapeutic promise of promoting opposing M2 responses.

## Classic Molecular Mechanisms Modulating Macrophage Phenotype

**Figure 1:**
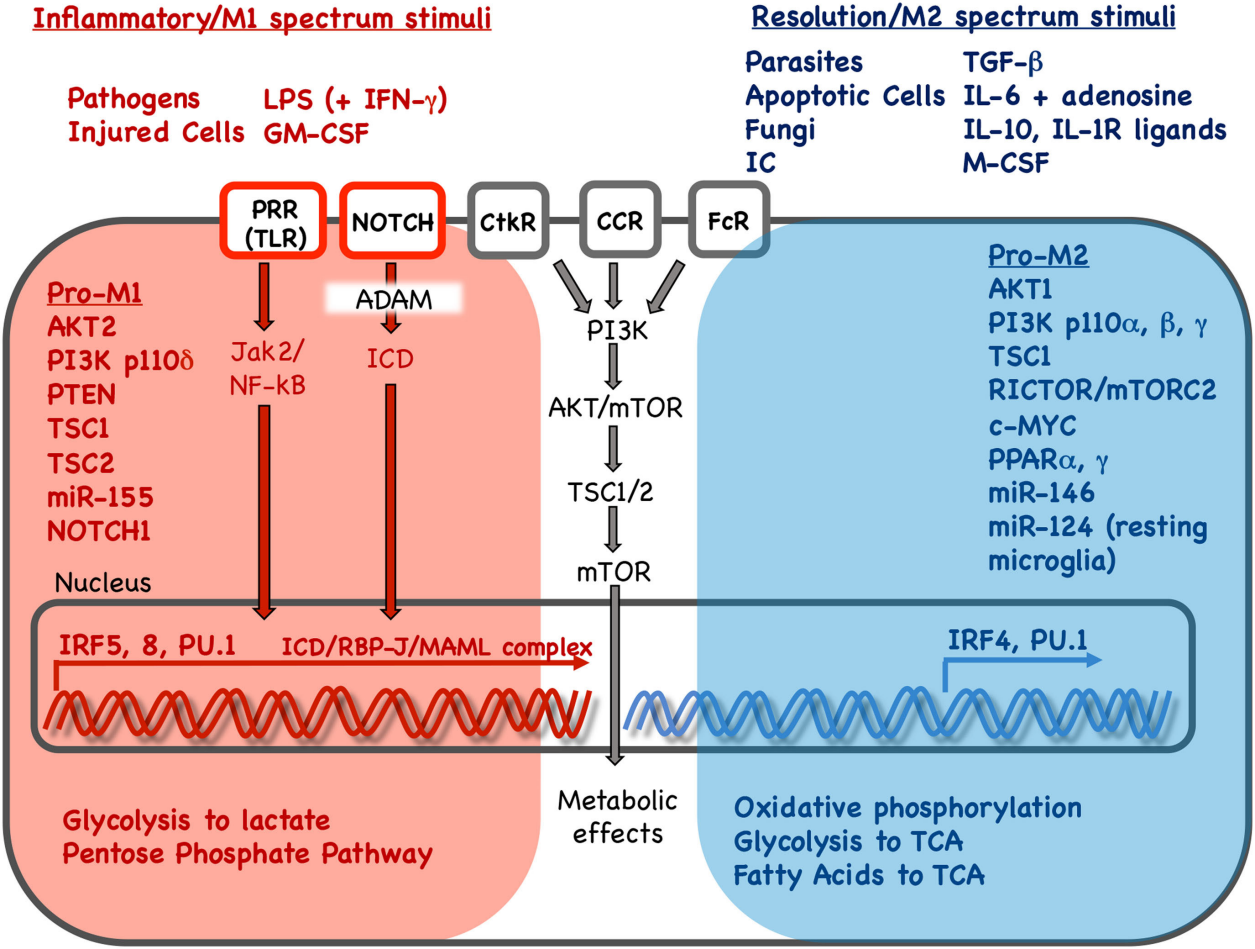
Key stimuli and molecular pathways in inflammatory vs. resolution phenotype in mononuclear phagocytes. Inflammatory and resolution macrophage phenotype results as external stimuli are integrated *via* signaling pathways to drive phenotype-supporting transcriptional programs and cellular metabolism. Red and blue color indicates pathways, stimuli, transcription factors and metabolic processes associated with inflammatory or resolution phenotype, respectively. Inflammatory phenotype is induced or promoted by pathogens, injured cells and *in vitro* stimuli. In contrast, resolution spectrum phenotypes are induced or promoted by parasites, fungi, apoptotic cells, immune complexes and other cytokine/growth factor stimuli. Pathogen or injury signals sensed *via* pathogen-recognition receptors (PRR) such as Toll-like receptors (TLR) result in Janus activated kinase (Jak)2 and nuclear factor kappa B (NF-κB) activation. Signals received *via* Notch receptors, cytokine receptor (CtkR), chemokine receptor (CCR), and Fc receptor (FcR) stimulation are also integrated, defining gene expression and downstream metabolic reprogramming. Interferon regulatory factors (IRFs) 5 and 8 promote inflammatory gene expression while IRF 4 promotes resolution phenotype genes. Gene expression promotes changes in nutrient uptake and metabolic pathways that support inflammatory or resolution macrophage phenotype. LPS, lipopolysaccharide; IFN-γ: interferon-γ; GM-CSF, granulocyte monocyte colony stimulation factor; IC, immune complexes; TGF-β, transforming growth factor-β; IL, interleukin; IL-1R, interleukin 1 receptor; M-CSF, monocyte colony stimulation factor; PI3K, phosphoinositide 3 kinase; AKT, serine threonine kinase; mTOR, mammalian target of rapamycin; PTEN, phosphatase and tensin homolog; TSC, tuberous sclerosis complex; c-MYC, PPAR, peroxisome proliferator activated receptor; ADAM, A disintegrin and metalloproteinase; RBP-J, recombination signal binding protein for immunoglobulin kappa J region; MAML, mastermind-like; Rictor, rapamycin-insensitive companion of mTOR; TCA, tricarboxylic acid/Krebs cycle.

The distinct phenotypic features of inflammatory vs. wound-healing macrophages are controlled by a network of molecular pathways that relay environmental signals *via* signaling cascades to impact gene expression and cellular metabolism (see Figure 1 for a summary of molecular pathways that modulate macrophage phenotype). PI3K/AKT, NOTCH, PPARs, MYC, and IRFs have been known to modulate macrophage phenotype. Novel data demonstrating crucial roles for metabolism, RTKs, miRNA and epigenetic modifications will be discussed in a subsequent section.

### PI3K/AKT

The PI3K/AKT pathway is activated in response to environmental stimuli such as PAMPs, cytokines/chemokines and hormones to regulate cell survival, proliferation, and differentiation. This pathway plays a pivotal role in the activation phenotype of macrophages [for a thorough review, see Vergadi et al. (85)]. PI3K activates downstream kinase AKT that may exist as three different isoforms, namely AKT1, AKT2, and AKT3. AKT signaling is considered to be an activation dampening signal that controls NO and inflammatory cytokine production after TLR signaling (86–88) and promotes anti-inflammatory cytokines such as IL-10 (89, 90). However, AKT signaling is also required for normal M1 responses (91–93). The AKT1 and AKT2 isoforms play opposing roles in macrophage polarization. AKT1 KO macrophages show enhanced iNOS and IL-12 production and bacterial clearance (91, 94, 95). These effects were mediated by induction of the pro-M1 factor miR-155 (96, 97) that suppresses the target CCAAT/enhancer-binding protein beta CEBPβ (91, 95), a pro-M2 factor (98). In contrast, AKT2 deficiency has the opposite effect, resulting in macrophages that express CEBPβ and signature M2 markers such as arginase-1, YM1, REMLα, and the regulatory cytokine IL-10 (91, 99). AKT2 KO macrophages appear to adopt this phenotype *via* high levels of miR-146 (93), which has been associated with dampening of inflammatory responses *via* targeting of IRAK1, TRAF6, and IRF5 leading to suppression of TLR signaling in macrophages and microglia (100–103). Consistent with AKT signaling dampening inflammation, *Akt3* gene deficient mice suffered more severe disease in the murine EAE model of MS, an effect mediated by both peripheral macrophages and microglia (104).

Downstream, the PI3K/AKT pathway regulates cellular metabolism *via* the tuberous sclerosis (TSC)/mammalian target of rapamycin (mTOR) pathway. This is interesting in light of the prominent role metabolism plays in determining macrophage polarization (see metabolism section below). Some data suggest that mTOR signaling inhibits M1 and promotes M2 polarization (105–108). In contrast, other results are more consistent with mTOR signaling promoting M1 polarization (105, 106, 109–112). However, further clarity on the precise roles of TSC and *mTORC1* and *mTORC2* gene isoforms on metabolism and phenotype is required.

In summary, AKT signaling relays diverse extracellular signals to engage the metabolic regulator mTOR pathway. While AKT signaling can activate inflammatory responses, it is essential for promoting a dampening response, thereby promoting resolution.

### Notch

Notch signaling controls embryonic development and differentiation in multiple tissues and organs. Notch receptors NOTCH 1-4 are expressed on the cell surface, where they bind Jagged (JAGGED1, JAGGED2) or Delta-like (DLL1, 3, 4) family ligands on neighboring cells. Binding triggers A disintegrin and metalloproteinase (ADAM)/γ-secretase-catalyzed release of the Notch receptors intracellular domain (ICD), allowing ICD translocation to the nucleus, where it heterodimerizes with recombination signal binding protein for immunoglobulin kappa J region (RBP-J). While RBP-J normally acts as a corepressor recruiter, the ICD/RBP-J complex promotes gene expression *via* recruitment of mastermind-like coactivator (MAML) (113, 114). In murine macrophages, increased expression of NOTCH1, NOTCH2, and Notch ligands DLL4 and JAGGED1 has been observed in response to inflammatory cues such as LPS, IFN-γ, or IL-1β (115, 116). NOTCH/RBP-J signaling in macrophages results in enhanced NF-κB signaling and induction of pro-M1 transcription factors IRF1 and IRF8 that in turn drive expression of multiple classical activation genes (115–121). Accordingly, reduced levels of inflammatory cytokines IL-6, IL-12, and IFN-γ are observed in response to LPS + IFN-γ in macrophages deficient in Notch1 or treated with the γ-secretase inhibitor DAPT (122, 123). Notch signaling also increases M1 phenotype by modulating glucose flux to the tricarboxylic acid (TCA) cycle, respiratory chain components and reactive oxygen species (ROS) generation (124). Microglial responses are similarly impacted by NOTCH signaling (125–127). Macrophage-specific *Notch* gene deficiency or γ-secretase inhibitor DAPT treatment suppress clinical disease in *in vivo* disease models, including EAE (126, 128, 129). Overall, NOTCH signaling appears to have a pivotal role in the development of pathogenic macrophage responses and therapeutic strategies that target NOTCH signaling may be beneficial in inflammatory diseases, including MS.

### Peroxisome Proliferator Activated Receptors

Peroxisome proliferator activated receptors (PPARs) are nuclear hormone receptors that act as transcription factors and play important roles in development, differentiation and metabolic regulation (130). PPAR ligands include fatty acids, prostaglandins (PG) such as PG J2 and leukotrienes (LT) such as LT B4 (131). PPARs heterodimerize with the retinoid X receptor (RXR) and bind DNA, modulating target gene transcription. There are three PPAR receptors, PPAR α, β/δ, and γ. Stimulation of macrophages with M2 stimuli such as IL-4 and IL-13 induces PPARγ and PPARβ/δ, which are necessary to stabilize M2 phenotype (132, 133).

PPARα activation has been linked to anti-inflammatory innate immune responses in macrophages. PPARα agonists have therapeutic activity in several inflammatory disease models [reviewed in Ref. (134), including EAE (135)]. Similarly, PPARα deficient mice suffer from worsened EAE disease (136). PPARα activation has also been shown to promote regulatory macrophages and mediate microbiota/gut homeostasis (137).

PPARγ activation has long been known to promote M2 polarization and suppress inflammatory cytokines in mouse and human macrophages (138–140). Activation of PPARγ with the flavonoid apigenin suppresses M1 macrophage inflammatory cytokine IL-1β and iNOS expression and promotes expression of alternatively activated phenotype markers by modulating NF-κB signaling (141). Similar suppression of LPS-induced inflammatory microglia has been reported *via* increased PPARγ signals (142). Accordingly PPARγ agonists suppress CNS neuroinflammation in the EAE model (143).

PPARβ/delta is thought to play an anti-inflammatory role, although immune activating effects have also been reported. For example, induction of M2 polarization by IL-4 and IL-13 is dependent on PPARβ/δ (144, 145). In addition, PPARβ/δ agonists suppress intestinal inflammation and EAE (146–148). However, studies in human monocyte-derived macrophages have shown that PPARβ/δ agonists both suppress inflammatory cytokines and suppress cytotoxic T cell inhibitory molecules PD-1L and IDO (149).

In summary, PPAR nuclear receptors are activated by resolution phase lipid mediators, promoting CNS macrophage and microglia phenotype switching toward resolution. Therefore, PPARs stand out as potential therapeutic targets in neuroinflammatory disease.

### c-Myc

c-Myc is a transcription factor that modulates cellular survival and proliferation and metabolism, with important roles in angiogenesis, tumorigenesis, and immune responses (71, 150). c-MYC was found first to be induced by M2 stimuli such as IL-4 and IL-13 (71). In human macrophages, c-MYC translocates to the nucleus and controls the expression of half of the M2-associated genes (71). Human M2 markers SCARB, ALOX15, and MRC1 are directly promoted by c-MYC while others are indirectly induced (71). C-MYC also promotes STAT6 and PPAR-γ expression, further stabilizing M2 phenotype (71). In mice, c-Myc has also been found in human tumor-associated macrophages (TAM) (151) and transcriptional profiling of murine BMDM has demonstrated that c-Myc is also a selective marker of murine M2 macrophages (47). c-Myc expression correlated with detectable Egr2 protein, specifically labeling M(IL-4) but not M (LPS + IFN-γ) BMDM macrophages (47).

The exact role of c-MYC in M2 macrophages is not entirely clear. c-MYC may influence macrophage proliferation, which is consistent with the loss of proliferation observed during M1 macrophage stimulation (152). A pivotal role of c-MYC may be to metabolically program the macrophage. While HIF-1α induction in M1 macrophages promotes use of glucose *via* aerobic glycolysis to yield lactate and produce ROS, c-MYC expression in M2 macrophages may allow glycolytic activity necessary for M2 differentiation (153), providing additional sources of fuel for TCA cycle/oxidative phosphorylation (152). To summarize, c-MYC is gaining recognition as a mouse to human-conserved pro-M2 transcription factor. While much research is needed to understand c-MYC’s mechanistic actions, its connections to metabolic regulation of phenotype provide an intriguing area for exploration.

### Interferon Regulatory Factors

Interferon regulatory factors are transcription factors that are activated in response to cytokines, *via* JAK/STAT signaling, and/or PAMPS and play important roles in innate and adaptive immunity. There are nine IRF family members, named IRF1-9 (154), which modulate macrophage phenotype. Our current understanding is that IRF1, 5 and 8 promote classical activation while IRF4 promotes alternative activation (155).

IRF5 is strongly induced by LPS and IFN-γ and GM-CSF stimuli and plays a prominent role in M1 activation. IRF5 interacts with RelA to bind target gene loci (156), resulting in enhanced IL-12 and IL-23 (157). IRF5 also promotes M1 polarization by association to MyD88 (158). IRF5 variants have been linked to MS (159), possibly *via* enhanced inflammatory activation. Suppression of IRF5 in EAE, *via* inhibition of the Aurora Kinase A, reduced inflammatory cytokines and improved clinical disease (160). Another IRF, IRF1, also contributes to M1 phenotype *via* induction of iNOS and IL-12 (161). IFN-γ stimulation of macrophages induces Batf2, which was shown be an M1-specific factor than interacts with IRF1 to induce Nos2, Tnf-α and Ccl5 (162). IRF8 contributes to M1 phenotype by activating IL-12 transcription in cooperation with IRF1 (163). The clinical relevance of IRF8 is highlighted by the link between IRF8 variants and MS (164, 165). Mice with a myeloid-specific deletion of IRF8 are resistant to EAE. IRF8 activates microglia and drives an IL-12 and IL-23 rich environment that promotes Th1 and Th17 responses (166). In contrast, IRF4 is instead a major mediator of M2 polarization (167). IRF4 inhibits pathogen sensing *via* suppression of MyD88 signaling (167, 168) and collaborates with histone deacetylase Jumanji D3 (Jmjd3) to promote expression of M2 genes Arg1, CD206, Ym1, and Fizz1.

Overall, IRFs essentially link cytokine and PAMP extracellular stimuli to signaling that enhances (IRF1, 5, 8) or suppresses (IRF4) inflammatory transcriptional programs. The association of IRFs to MS risk and EAE disease by impacting macrophages and microglia highlight the importance of IRFs and their potential as therapeutic targets.

## Emerging Pathways

### MicroRNA

MicroRNA are small (~22 nucleotides) RNAs that are regulated in response to inflammatory signals, modulating macrophage and microglia activation and phenotype. The biogenesis of miRNA starts with transcription of a primary (pri-miRNA) transcript that undergoes several processing steps (169). The first involves Drosha/DCGR8 complex cleavage to generate a double-stranded hairpin precursor termed pre-miRNA. This is followed by pre-miRNA export to the cytoplasm, where Dicer eliminates the hairpin yielding a miRNA–miRNA duplex. One miRNA strand is then loaded onto the RISC complex for binding to target mRNA transcript. miRNA generally suppresses target gene expression *via* either induction of mRNA degradation or inhibition of translation (170).

In the context of inflammatory stimuli, miRNA modulate their expression and macrophage polarization (see Table 2). The importance of miRNA in macrophage/microglia polarization in now well documented. miRNA such as miR-155, miR-146, miR-101, miR-21 and let-7 family are induced in response to inflammatory stimuli while miR-223, miR-125b, and the miR-23/27a/24-2 cluster are instead downregulated. Among these miRNAs, miR-155 stands out as a miRNA necessary for inflammatory phenotype. miR-146 is instead induced by inflammatory stimuli to dampen the inflammatory response. These two miRNAs and the pathways they control are therefore discussed in detail below, together with a summary of the contributions of other miRNAs to pathways that modulate macrophage and microglia phenotype.

**Table 2:** Summary of miRNAs linked with M1 or M2 phenotype.

M1 stimulated	
miRNAs with proinflammatory effects	
microRNA	Modulated Pathways
miR-92	*Mkk*γ
miR-101	*Mkp1*
miR-105	*Tlr2*
miR-155	*Inpp5d/SHIP-1, Cebpb, Creb, Bcl6, Sfp1, IL-13Ra, SOCS1 and Sfp11, MafB*, and *Tspan14*
miR-223	*Pknox1*

**miRNAs with anti-inflammatory effects**	

**microRNA**	**Modulated Pathways**

miR-21	*NFkB, Il6, Tnfa, Tlr4*, and *Tlr8*
miR-101	*Dusp1*
miR-125b	*Irf4*
miR-146	*Irak1, Traf6, Irf5, Tlr4*, and *Stat1*
miR-223	*Cebpb, Rasa1*, and *Nfat5*

**M2 stimulated**	

**miRNAs with proinflammatory effects**	

**microRNA**	**Modulated Pathways**

miR-26	*Atf2* and *Tnf*
miR-27	*Trc4, Irak4, Il6, Il1*β, *Tnf*α, *Nos*
miR-let7b	*Tlr4, Tnf*α
miR-let7i	*Tlr4*

**miRNAs with anti-inflammatory effects**	

**microRNA**	**Modulated Pathways**

miR-23/27a/24-2	*Jak1*/*Stat6, Irf*/*Ppar*γ, *Tlr4*, and *Irak4*
miR-21	*Arg1, Il4*α, *Mrc1*, and *Pge2*
miR-146	*Irak1, Traf6, Irf5, Tlr4*, and *Stat1*
miR-181	*Il1a, Il6*

miR-155 is robustly induced in CNS inflammatory conditions such as MS and spinal cord injury (171–174). Several cells in the CNS, including macrophages (175), microglia (176), astrocytes (177), and neurons (178) may express miR-155. miR-155 is the most highly upregulated miRNA after exposure to a range of inflammatory stimuli including M [LPS + (IFN-γ)] conditions in both murine/human macrophages (96, 173, 179–183) and microglia (173, 176, 184). In contrast, exposure to alternatively activating stimuli such as IL-4 does not induce miR-155 (96, 185). The quick and swift induction of miR-155 suggests that miR-155 plays a crucial role in determining the classically activated macrophage phenotype. In support of this hypothesis, miR-155 delivery into macrophages or microglia *via* exosomes enhances inflammatory gene expression, including IL-6 and IL-12 (186). miR-155 in microglia also modulates phenotype *via* suppression of SOCS-1 and enhancement of NO and cytokine production (176). So, what are the global effects of miR-155 on M1 phenotype? Transcriptional profiling in miR-155 KO macrophages exposed to M(LPS + IFN-γ) conditions reveals that approximately half of the M(LPS + IFN-γ) signature is miR-155 dependent (96). These results indicate that miR-155 is required for full expression of inflammatory macrophage signature. Among the most impacted genes, inflammatory cytokines such as IL-1β, IL-6 and IL-12, inflammatory enzymes such as iNOS and costimulatory molecules such as CD86 and CD40 and adhesion and migration molecules such as CD49E and CCR7 stand out (96, 97). These data are consistent with clinical improvement of EAE, SCI and stroke in animals deficient in miR-155 or treated with miR-155 antisense oligonucleotide inhibitors (171, 187). Since miRNAs typically suppress targets rather than promote them, the inflammatory gene activation effects of miR-155 are expected to be mediated by suppression of deactivating genes. Validated miR-155 targets include transcripts of the *Inpp5d, Cebpβ, Creb, Bcl6, Sfp1, IL-13Rα, Socs1, Sfp11, MafB*, and *Tspan14* genes. We found that the transcripts of target genes *Inpp5d, Ptprj, MafB* and *Tspan14* inversely correlated with miR-155 (96). Targeting of *Inpp5d* by miR-155 promotes AKT signaling (188) in murine macrophages. In addition, CEPBβ has been shown to promote alternatively activated genes IL-10, IL-13Ra, arginase-1, RELMα (98) and targeting by miR-155 may suppress M2 phenotype. This is in contrast to the later finding that CEBPβ targeting by miR-223 is essential to prevent inflammatory macrophage development and colitis (189). MAF suppresses IL-12 and promotes IL-10 production in macrophages (190, 191) and its inactivation by miR-155 may be required to initiate the inflammatory gene expression program.

Although miR-146a/b are coinduced with miR-155 in response to M1 stimuli (192), they have opposite effects. While miR-155’s role is to release the brake on inflammation, miR-146 instead sets off a series of events that will eventually dampen inflammation. miR-146 targeting of TRAF6, IRAK1, TLR4, and STAT1 appear to mediate some of these effects by limiting responsiveness to inflammatory stimuli (100–102). Similar results have been observed in microglia, where miR-146 has been shown to promote M2 phenotype by targeting IRAK1/TRAF6 (103).

Other miRNAs modulated with macrophage/microglia phenotype include miR-124, miR-125b, miR-223, miR-101, miR-21, the let-7 family and the miR-23a/27a/24-2 cluster. Effects on phenotype appear to be achieved through targeting to JAK/STAT, NF-kB, or MAPK pathways and CEBP, PPAR, or IRF family transcription factors as further described below or in Table 2.

The CEBP family of transcription factors is targeted by miR-124, miR-223, and let-7 family miRNAs in macrophages and microglia. miR-124 and miR-223 target CEBPβ, which promotes macrophage development (98) and both inflammatory (193–196) and alternatively activating cytokines (197, 198). miRNA-124 is expressed in microglia, promoting a resting phenotype, but not in peripheral origin macrophages/monocytes (199). miR-124 treatment suppresses microglia and macrophage activation, T cell infiltration and clinical disease in EAE and other CNS inflammatory models, suggesting it may beneficial in MS (199, 200). miR-223 maintains a deactivated state through targeting of CEBPβ (189). miR-let-7c is associated with less inflammatory GM-CSF induced macrophages, where it suppresses the pro-inflammatory TF CEPBδ (201). miR-let-7i (202) and miR-let-7b (203) have also been shown to dampen inflammation by suppression of TLR4 expression.

Many miRNAs modulate macrophage phenotype by dampening or promoting the NF-kB pathway that proinflammatory stimuli induce. For example, miR-125b promotes activated microglia phenotype by suppressing A20/NF-κB signaling (204). miR-let-7f also targets the NF-κB negative regulator A20, resulting in enhanced IL-1β and TNF-α (205). In addition, let-7b also acts as a TLR7 agonist in microglia, activating TLR signaling and downstream NF-kB activity and leading to inflammatory microglia and neurodegeneration (206). Consistent with this, let-7b correlates with TNF-α production in tumor-associated macrophages (207).

In summary, miRNAs are now established as important regulators of macrophage and microglia that modulate neuroinflammation and neurodegeneration. miR-155 and miR-146 have robust pro- and anti-inflammatory roles, respectively, that may be therapeutically harnessed in MS and other neuroinflammatory diseases.

### Metabolism

**Figure 2:**
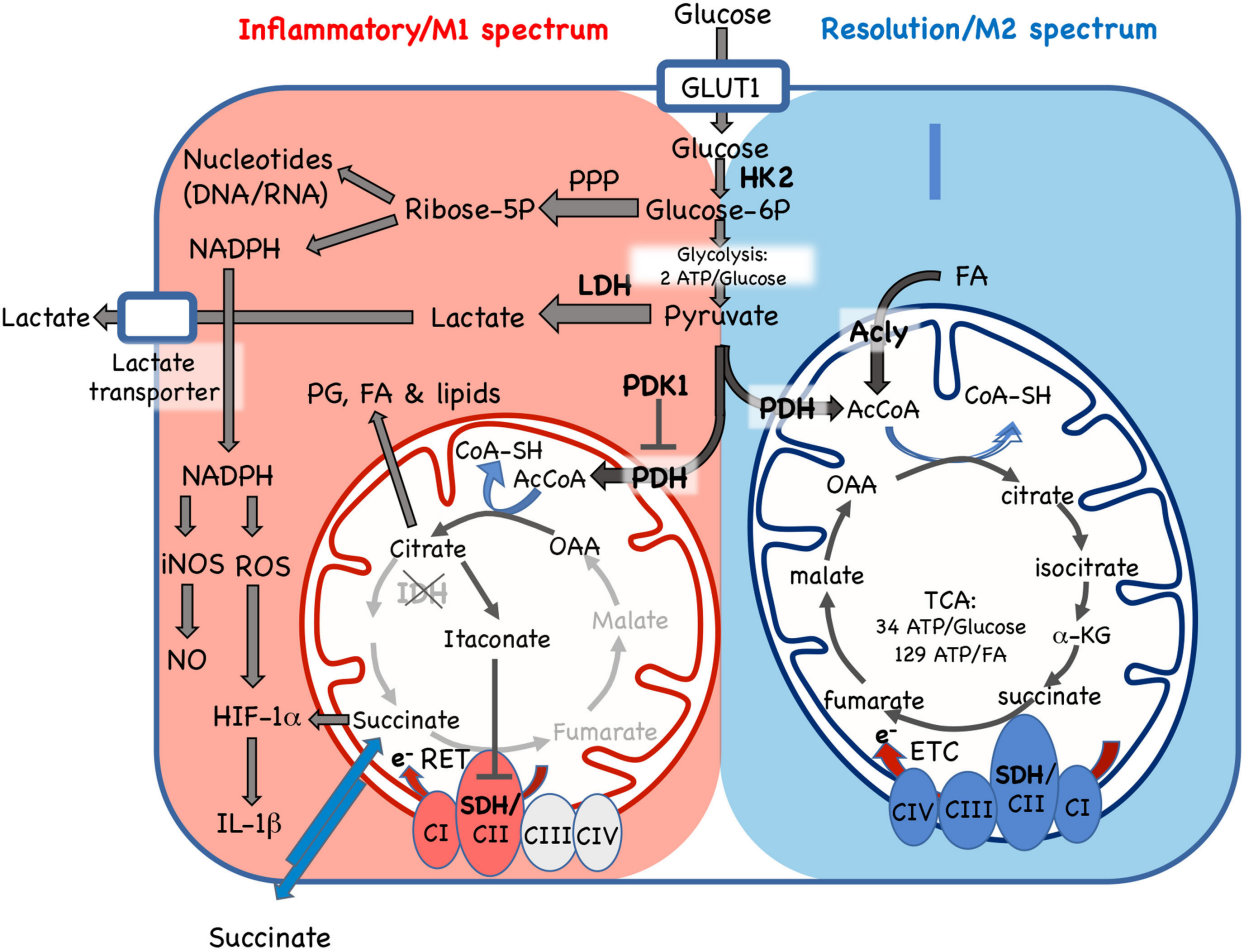
Cellular metabolic pathways driving mononuclear phagocyte inflammatory vs. resolution phenotype. Glucose is essential for both inflammatory and resolution macrophage phenotypes. In inflammatory macrophages, glucose is largely processed to yield lactate *via* aerobic glycolysis. Another major pathway in M1 spectrum macrophages is conversion to ribose-5P *via* the pentose phosphate pathway (PPP) for synthesis of nucleotides and NADPH, which supports nitric oxide (NO), reactive oxygen species (ROS), and IL-1β production. In resolution macrophages, the major fate of glucose is the TCA/Krebs cycle *via* pyruvate dehydrogenase (PDH)-catalyzed conversion of pyruvate to AcetylCoA (AcCoA). The TCA cycle in resolution macrophages is also fed by fatty acids (FA) *via* the ATP-citrate lyase (Acly) enzyme and promotes forward electron transport chain (ETC), from C1 to CIV, for ATP generation. In contrast, while some glucose enters the mitochondria in inflammatory macrophages, where it is converted to citrate, the TCA cycle is broken, with stops at the isocitrate dehydrogenase (IDH) and succinate dehydrogenase (SHD) steps. Citrate accumulation results in itaconate production, which inhibits SDH, and also promotes prostaglandin (PG), lipid and FA synthesis. In inflammatory macrophages, these blocks promote reverse electron transport chain (RET) from CII to CI. GLUT1, glucose transporter 1/SLC2A1; HK2, hexokinase 2; Glucose-6P, glucose-6-phosphate; LDH, lactose dehydrogenase; PHK1, pyruvate dehydrogenase kinase 1; AcCoa, acetyl coenzyme A; TCA, tricarboxylic acid/Krebs cycle; a-KG, a-ketoglutarate; CI-IV, ETC complexes I–IV, SDH, succinate dehydrogenase; OAA, oxaloacetate; e^-^, electrons; RET, reverse electron transport chain; iNOS, inducible nitric oxide synthase; HIF-1α, hypoxia inducible factor-1α. Enzymes are indicated by bold font.

Metabolism is taking center stage in our understanding of pathways that modulate macrophage phenotype. While it was understood that inflammatory macrophages necessitate metabolic adaptations to offset high energy requirements, the realization that metabolism in fact *determines* inflammatory or regulatory phenotype is possibly a paradigm shift. Since metabolic pathway choice depends on enzyme activity, it provides interesting new (or repurposed) therapeutic strategies for inflammatory disease, further discussed in section 8. For a summary of how metabolic pathways differ in M1 and M2 macrophages, refer to Figure 2.

Differences in amino acid metabolism have long been observed among macrophage phenotypes (208, 209). While M1 macrophages upregulate iNOS to convert arginine to NO for microbial killing, M2 macrophages induce arginase-1 and catabolize arginine to produce polyamines and proline for collagen biosynthesis. Additional differences in ATP generation (glycolysis vs. mitochondrial oxidative phosphorylation), pentose phosphate pathway activity, and TCA use have recently been demonstrated (210).

- ATP generation. Most tissues, including M2 macrophages, use mitochondrial oxidative phosphorylation (34 ATP/glucose or 129/palmitic acid) as a source of energy. In contrast, M1 macrophages rely on aerobic glycolysis to lactate (2 ATP/glucose) for energy generation (211). Aerobic glycolysis is also known as Warburg metabolism and this phenotype has also been observed in cancer cells (212).

Why a lower ATP output pathway would be chosen by M1 macrophages is intriguing. Glycolysis may provide rapid ATP production. However, new evidence that NO production in M1 macrophages strongly inhibits oxidative phosphorylation by impairing the electron transport chain (213) provides an alternative explanation for the need to use an alternative pathway for ATP generation.

While M1 macrophages have increased glycolytic activity as compared to M2 macrophages, M2 macrophages also depend to some extent on access to glucose and its oxidation *via* glycolysis. Although the TCA cycle in M2 macrophages was thought to be mostly fueled by fatty acids (210), recent work by has shown that an active glycolysis pathway is essential for TCA/oxidative phosphorylation and M2 phenotype (153).

- Pentose phosphate pathway (PPP). The PPP directs some glucose-6P away from the glycolysis pathway and into generation of ribose-6P and derivatives. This pathway yields nucleotides to support DNA replication and RNA transcription and NADPH for ROS and NO generation. In addition to increased glycolysis, high PPP activity is characteristic of M1 macrophages (214).
- Krebs/TCA cycle. M2 macrophages rely almost exclusively on ATP generation *via* oxidative phosphorylation coupled to an intact TCA cycle (213, 215, 216). In contrast, in M1 macrophages the TCA cycle is “broken” at two steps: citrate to α-ketoglutarate and succinate to fumarate (217, 218). Reduced isocitrate dehydrogenase activity leads to citrate accumulation in M1 macrophages. Citrate supports M1 phenotype by promoting FAS and membrane biosynthesis, prostaglandin and itaconate production. Itaconate inhibits succinate dehydrogenase causing the second break in the cycle and succinate accumulation (219). Succinate stabilizes HIF-1α and promotes IL-1β production in LPS stimulated macrophages (217, 218). The second break in the TCA cycle is linked to a reversal in electron transport chain direction that fuels an increase in mitochondrial membrane potential and supports classic M1 NO and ROS generation (217).

In summary, M1 and M2 macrophages use opposing metabolic pathways to fulfill energy and biosynthetic requirements. M1 macrophages favor glycolysis to lactate and PPP pathways to provide energy and support NO and ROS generation. M2 macrophages instead rely on TCA cycle for ATP generation. This metabolic dichotomy is not a consequence of M1 or M2 phenotype, but rather a requirement for either phenotype, therefore providing exciting opportunities for therapeutic targeting.

### Receptor-Tyrosine Kinases

Receptor-tyrosine kinases have been proposed to fine-tune macrophage function in immunity and tissue homeostasis. Macrophages are known to express RTKs within three families of RTKs, namely platelet-derived growth factor receptor (PDGFR), the AXL/TYRO3/MERTK family, and the RON superfamily. Colony stimulation factor receptor 1 (CSF1R) is involved in macrophage development and is a member of the PDGFR superfamily (220). The PDGFR family receptors are characterized by 5 Immunoglobulin (Ig)-family domains and a kinase domain. The TAM (TYRO3/AXL/MERTK) family is instead characterized by two Ig-like domains, two fibronectin III repeats and a Kinase domain (221, 222). Finally, the human RON receptor (STK in mouse) is a member of the MET family of RTKs (223, 224). Ligation of RTKs to ligands, such as M-CSF to CSF1R, apoptotic cell phosphatidyl serine (PtdSer) *via* grown arrest specific 6 (Gas6) and Protein S bridging to TAM family RTKs and macrophage-stimulating protein (MSP) to STK results in activation of kinase activity (225–227).

The active form of STK ligand MSP is generated *via* the coagulation cascade (228) and has a crucial role in the response of macrophages to inflammatory cytokines and LPS. MSP dampens NO and PGE2 production *via* suppression of iNOS and COX-2 expression (229–231). TAM receptors mediate apoptotic cell removal after PtdSer is recognized *via* Gas6 or Protein S, activating MerTK and reducing TNF-α and LPS responsiveness (232). NO activity, IL-12 production and MHCII expression are also controlled by MSP: MSP exposure prior to LPS + IFN-γ activation inhibits these signature M1 factors *via* arginase-1 induction (233). Consistent with the proresolving role of RTKs, macrophages from *Tyro3, MerTK*, and *Axl* triple KO mice display enhanced IL-12, MHCII, and costimulatory molecules in response to LPS (234).

The AXL RTK is also induced in mouse and human macrophages by type I IFNs and TLR3 stimulation (235). This induction may signal enhanced apoptotic cell removal needs during inflammation. The Rothlin group added a relevant layer to the physiologic role of TAM RTKs AXL and MERTK in resolution of immune responses to infection and promotion of tissue repair (60). They found that including apoptotic cell ligands for TAM RTKs strongly enhanced the expression of anti-inflammatory and tissue repair genes, including RELMα, CHI3L3, FN1, and EAR2 in response M2 (IL-4) stimuli (60) *in vitro*. Such signaling was essential to dampen inflammation and allow tissue repair in thioglycollate and helminth infection models. Their results implicate that apoptotic cell sensing by AXL and MERTK in the presence of IL-4 responses drives anti-inflammatory and tissue repair programs in macrophages. It is interesting to speculate whether the failure of most interventions to enhance M2 phenotype during injury (236, 237) may stem from deficiencies in these pathways. If so, small molecule-mediated stimulation of these pathways during chronic CNS inflammation may be a promising therapeutic strategy to promote proregenerative macrophages/microglia.

In summary, RTK activity is largely stimulated by resolution mediators, such as MSP and apoptotic neutrophil PtdlSer in late stages of acute inflammation. These signals effectively suppress inflammatory responses and promote and amplify a resolution macrophage phenotype switch. These findings highlight a physiologically relevant pathway for inflammation resolution that may be therapeutically harnessed in neuroinflammatory disease.

## Therapeutic Strategies for Macrophage/Microglia Phenotype Reprogramming

Increased understanding of the molecular pathways that promote inflammatory and resolution phenotypes in macrophages and microglia provides therapeutic targets for inflammatory diseases, including the autoimmune disease MS. Due to the increasing importance of metabolic reprogramming in macrophage phenotype, we will focus our discussion on the current understanding of how available metabolic reprogramming drugs may impact macrophage phenotype and MS.

Dichloroacetate (DCA) is an inhibitor of PDK1, a kinase that in turn suppresses PDH. DCA treatment therefore increased PDH activity, shifting cellular metabolism toward the TCA cycle/oxidative phosphorylation and promoting a proregenerative M2 resolution phenotype (238). Consistent with a shift from M1 to M2 phenotype in macrophages and microglia, treatment with DCA suppressed clinical disease scores and T cell infiltration in the EAE model of MS (239).

Dimethylfumarate (DMF) is an approved oral drug for MS treatment that has been shown to efficiently reduce relapse rate and disease progression (240). DMF is thought to exert its therapeutic effects *via* activation of the Nrf2 pathway and induction of the antioxidant response. Could DMF’s metabolite monomethyl fumarate enter the TCA cycle at the fumarate step, thereby modulating oxidative phosphorylation? Consistent with this scenario, increased TCA cycle intermediates malate, fumarate and succinate are observed in DMF-treated oligodendrocytes (241). However, it is unknown whether similar effects occur in macrophages and microglia. Microglia and myeloid cell pretreatment with DMF does however reduce NO and inflammatory cytokine production (242, 243), although these effects have not been recapitulated in DMF-treated EAE mice (244). Similarly, increased superoxide generation has been observed in fumarate-treated monocytes (245), indicating that DMF’s effects may depend on timing and context.

Many of the current drugs targeting metabolic pathways are currently approved for type II diabetes and metabolic syndrome therapy. One of the first drugs for type II diabetes, the biguanide family metformin, inhibits AMPK and complex I of the electron transport chain (ETC) (246). Treatment of LPS-activated macrophages with metformin suppresses NO and IL-1β production while increasing IL-10 (247). This effect was due to inhibition of complex I, which is necessary for respiratory electron chain in M1 macrophages (247). *In vivo* metformin treatment reduced inflammatory cytokine production, reduced Th17 responses and enhanced Tregs, overall ameliorating EAE disease (248, 249). The common use of metformin for metabolic syndrome has allowed to test whether it is beneficial in MS. In an open-label study of 50 obese MS patients, the 20 patients who received metformin had decreased new or enlarging T2 and gadolinium-enhancing lesions (250, 251). These clinical effects were accompanied by significant decreases in inflammatory Th1 and Th17 cells but significant increases in Tregs in metformin-treated patients (250).

Pioglitazone and rosiglitazone are antidiabetic drugs from the thiazolidinedione family that act as full agonists of PPARγ, modulating multiple metabolic processes, particularly lipid and glucose metabolism (130, 252). A smaller number of patients in the metformin study above were treated with pioglitazone and similarly benefited from reduced lesions and shifts from inflammatory to regulatory T cell responses (250). These clinical effects are consistent with previously reported suppression of microglial activation and clinical disease in the EAE model (253, 254).

## Concluding Remarks

Recent studies have provided us with a more thorough and insightful understanding about how steady-state and acute environmental signals are integrated by macrophages and their microglial counterparts to maintain optimal neurologic function, eliminate infection and resolve injury or inflammation. Besides PRR-induced Jak2/NF-κB signaling, Notch receptor signaling and PI3K/AKT2/miR-155 signaling contributes to M1 phenotype. In contrast, PI3K/AKT1/miR-146 and apoptotic cell-induced RTK signaling promotes M2 phenotype. These effects are achieved through IRF-mediated control of gene expression, leading to changes in how cellular nutrients are used *via* metabolic pathways. M1 macrophages turn to aerobic glycolysis/Warburg metabolism and high PPP activity for energy production and simultaneously promote NO, ROS, and IL-1β expression. Interestingly, NO inhibits oxidative phosphorylation, which may explain the need for TCA-independent sources of energy in M1 macrophages. In contrast, M2 macrophage polarization and function is entirely dependent on TCA/oxidative phosphorylation, although glucose is also essential for M2 phenotype. Links between classic pathways known to affect macrophage phenotype and metabolism are starting to emerge. The PI3K/AKT pathway activates mTOR signaling, a major regulator of glucose metabolic pathways in the cell. Similarly, PPARγ is another major regulator of macrophage phenotype and lipid/glucose metabolism. Finally, c-MYC is an important mediator of M2 phenotype in murine and human macrophages that modulates glucose fuel utilization. The requirement of active metabolic pathways for specific macrophage phenotypes constitutes a paradigm shift in the field, away from a mere supporting role. Perhaps more importantly, it provides an opportunity for therapeutic modulation at the step where signaling pathways converge to determine phenotype. Novel and repurposed metabolic reprogramming drugs may provide promising alternative therapeutic strategies in MS and other neuroinflammatory disorders. So far, a study has shown excellent clinical MS responsiveness using metformin and pioglitazone in obese MS patients. Further studies are required to evaluate the efficacy of these treatments in the general MS population and understand how they impact macrophages and microglia phenotype. The field is ripe to address these questions and exciting basic knowledge and therapeutic opportunities lie ahead.

## Author Contributions

All authors: conception of idea, review of the literature, manuscript writing and editing.

## Conflict of Interest Statement

The authors declare that the research was conducted in the absence of any commercial or financial relationships that could be construed as a potential conflict of interest.
